# Evolution of community-associated MRSA: a 20-year genomic and epidemiological study in Region Örebro County, Sweden

**DOI:** 10.3389/fmicb.2024.1504860

**Published:** 2024-12-23

**Authors:** Jan Kekki, Annethe Thegel, Bianca Stenmark, Bo Söderquist

**Affiliations:** ^1^Clinical Genomics, Faculty of Medicine and Health, Örebro University, Örebro, Sweden; ^2^Department of Pathology and Clinical Genomic, Faculty of Medicine and Health, Örebro University Hospital, Örebro, Sweden; ^3^Department of Infection Control, Örebro University Hospital, Örebro, Sweden; ^4^Laboratory Medicine, Department of Clinical Research Laboratory, Faculty of Medicine and Health, Örebro University, Örebro, Sweden; ^5^Department of Laboratory Medicine, Clinical Microbiology, Faculty of Medicine and Health, Örebro University, Örebro, Sweden; ^6^School of Medical Sciences, Faculty of Medicine and Health, Örebro University, Örebro, Sweden

**Keywords:** *Staphylococcus aureus*, epidemiology, CA-MRSA, MLST, SCC*mec*, clonal complex

## Abstract

**Background:**

Methicillin-resistant *Staphylococcus aureus* (MRSA) has been an issue in healthcare since the 1960s. It was initially found only in healthcare facilities, but in the late 1990s it began to be seen with no healthcare connexion. The mechanisms of intercontinental and national spread are not fully understood, as sometimes novel outbreaks occur without any identifiable source or connexion to locally dominant clonal clusters.

**Methods:**

This study investigated the epidemiology and genomics of community-associated MRSA in Region Örebro County, Sweden, through 330 isolates collected between 2000 and 2019.

**Results:**

A shift in the dominant sequence type (ST) from ST80 to ST22 occurred in 2011–2019, along with an increase in the prevalence of STs belonging to clonal complexes CC5 and CC22. Both ST8 and ST80 isolates seemed to give way to emerging ST22 isolates, also indicated by the declining presence of the USA300 clone. The staphylococcal chromosomal cassette *mec* (SCC*mec*) type IV Remained dominant.

**Conclusions:**

The SCC*mec* type IV characteristic appears to be relatively geographically stable, possibly due to its low fitness cost and transductal capabilities. This warrants further studies of SCC*mec* type IV variant's survival mechanics as well as the effects of migratory flow on local epidemiology, in preparation for future possible outbreaks.

## Introduction

The opportunistic gram-positive bacterium *Staphylococcus aureus* is one of the most common infectious agents in humans. It can infect a wide range of areas including the skin, soft tissue, bones, and joints, potentially causing severe conditions such as pneumonia, prosthetic joint infection, and bacteraemia (Chambers and DeLeo, 2009; Cheung et al., 2021). Humans constitute a primary reservoir, with approximately 30% of the human population being carriers (Shopsin et al., 2000; Chen et al., 2013). Households, pets, and livestock have all been suggested to act as local reservoirs, and the food distribution chain and intercontinental travel have been described as possibilities for international spread; however, parts of community-associated methicillin-resistant *S. aureus* (CA-MRSA) epidemiology remains, as of yet, in need of further study (Pang et al., 2020, 2021; Zhu et al., 2021; Lee et al., 2018).

*S. aureus* has a predisposition for acquiring resistance to penicillin through the enzyme penicillinase, encoded by *blaZ*, and/or production of an alternate penicillin binding protein, penicillin binding protein 2A, encoded by *mecA* or *mecC*, also conferring resistance to penicillinase-resistant penicillins (McGuinness et al., 2017; Lakhundi and Zhang, 2018). In addition, *S. aureus* can develop resistance against other antibiotic groups, thus displaying multi-drug resistance. Methicillin-resistant *S. aureus* (MRSA) was initially considered predominantly a healthcare issue, as the first reports of MRSA in the 1960s were associated with hospitals and other healthcare facilities (Rountree and Freeman, 1955). Today, isolates connected to healthcare facilities are referred to as hospital-associated MRSA (HA-MRSA). Observations of MRSA in the community without any connexion to healthcare settings were first made in Australia in the 1980s (Nimmo and Coombs, 2008). Australia also recorded the first highly virulent strain of CA-MRSA in the 1990s, and CA-MRSA has since emerged across the world (Elston, 2007; Nimmo and Coombs, 2008). These isolates generally differ in genomic characteristics and virulence patterns from HA-MRSA; however, CA-MRSA has recurrently been known to invade hospitals (Berglund et al., 2005b; Turner et al., 2019; Sato et al., 2023).

Acquisition of the SCC*mec* element, the common genomic denominator of MRSA, results in resistance to most beta-lactam antibiotics. The *mec* gene is divided into types, A-C and E, where only class A and C has been found in *S. aureus*. It is harboured in the mec complex housing the *mec* gene and its regulatory components, five types of *mec* genes are known (A,B,C1,C2, and E) (Uehara, 2022). The *mec* complex is carried in a small chromosomal mobile genetic element, allowing for horizontal transfer by natural transformation, or by plasmid or a suspected ability of phage-mediated transduction, and is referred to as the staphylococcal cassette chromosome *mec* (SCC*mec*) [Katayama et al., 2000; International Working Group on the Classification of Staphylococcal Cassette Chromosome Elements (IWG-SCC), 2009; Scharn et al., 2013; Cheung et al., 2021]. At present there are 15 known types of the SCC*mec*, defined by the *ccr* complex, class of the *mec* complex, the integration site in the staphylococcal chromosome and presences of direct repeats flanking the integration site sequence [International Working Group on the Classification of Staphylococcal Cassette Chromosome Elements (IWG-SCC), 2009; Uehara, 2022; Wang et al., 2022]. CA-MRSA mostly carries the smaller variants of SCC*mec*, such as SCC*mec* IV and V, in contrast to HA-MRSA where the larger SCC*mec* elements are more prominent (Lakhundi and Zhang, 2018; Uehara, 2022). CA-MRSA displays similar phenotypic patterns in penicillin resistance as HA-MRSA, although the arrangement in the SCC*mec* cassette might differ, as well as resistance genes to other non-beta-lactam antibiotics and/or toxins and virulence factors [International Working Group on the Classification of Staphylococcal Cassette Chromosome Elements (IWG-SCC), 2009; Turner et al., 2019].

The phage-mediated leukotoxin known as Panton–Valentine leukocidin (PVL), encoded by *lukS/F -pv*, was a common trait of the USA300 clone, an outbreak strain in the United States in the early 2000s (Gillet et al., 2002; Boakes et al., 2011). It is debated whether this toxin can be considered as a virulence factor of CA-MRSA, resulting in a less favourable clinical outcome, as it has been linked to clinical phenotypes causing recurring skin and soft tissue infection and necrotizing pneumonia (Gillet et al., 2002; Lemaître et al., 2013; Glaser et al., 2016; McGuire et al., 2023). During the last decade one of the most dominant CA-MRSA sequence type (ST) in Europe is ST80, considered as a prominent carrier of PVL (Mairi et al., 2020). It was first identified in Greece in 1998, but has been traced back as early as 1993 in Denmark (Faria et al., 2005). It has been identified in at least 23 European countries. In the Middle East, ST22 and ST80 dominate, usually carrying SCC*mec* IV (Lakhundi and Zhang, 2018; Turner et al., 2019). In Asia, ST59 carrying SCC*mec* IV and V is predominant, but ST8, ST80, and ST22 carrying SCC*mec* IV are also common. A previous study in Region Örebro County on isolates from 1995 to 2010 showed that ST80 was dominant and exclusively found among CA-MRSA, while ST45 was the dominant ST among HA-MRSA (Berglund et al., 2005a). However, the geographical background of some of the clonal complexes (CCs) found suggested the beginning of an import of strains from epidemic areas outside of Sweden.

The aim of the present study was to examine the long-term epidemiology of CA-MRSA in Region Örebro County during the period 2000–2019, and to investigate whether immigration during the period had had any altering effects on the CA-MRSA epidemiology in this region.

## Method and material

### Case definition

Primary CA-MRSA isolates from Region Örebro County during 2000–2019 were identified using the definition of CA-MRSA from a previous study by Berglund et al. (2005b): “Community-acquired infection was recorded following identification of MRSA in an outpatient setting, or if a patient was positive for MRSA within 48 h of admission to a hospital, provided that the patient had no medical history of MRSA infection or colonisation, no hospitalisation, admission to a nursing home, dialysis or surgery during the previous year, and no permanent indwelling catheters or medical devices that penetrated the skin.” The isolate collection was divided into two cohorts according to the time of isolation: 2000–2010 and 2011–2019.

### Case identification

Essential epidemiological information has been collected in Region Örebro County since 2000, in accordance with the Communicable Diseases Act which governs contact tracing in Sweden, and stored in SmiNet, the contagious disease registration system administered by The Public Health Agency of Sweden (n.d). Data regarding the year of isolation and country of origin of each isolate were retrieved from SmiNet. Country of origin is determined during the contact tracing investigation, performed by the County Medical Officer for Communicable Disease Control.

All clinically identified CA-MRSA cases in Region Örebro County during 2000–2019 were included (*n* = 330). Isolates related to primary cases (e.g., intrafamily or outbreak-related) were excluded based on outbreak data recovered from SmiNet (The Public Health Agency of Sweden, n.d).

### Isolate collection

In Region Örebro County, clinically identified MRSA isolates are stored as per routine at the Department of Clinical Microbiology at Örebro University Hospital. Pure cultures were preserved at −80°C in preservation medium consisting of trypticase soy broth supplemented with 0.3% yeast extract (BD Diagnostic Systems, Sparks, MD, USA) and 29% horse serum (Håtunalab AB, Håtuna, Sweden) according to routine procedures.

### DNA extraction

In preparation for DNA extraction, isolates were cultivated on blood agar at 37°C overnight and treated with lysostaphin (Sigma-Aldrich, Saint-Louis, Missouri, USA) in a NaCl suspension before DNA extraction. DNA extraction from isolates was performed with automated magnetic bead extraction on a QIAsymphony instrument (Qiagen, Hilden, Germany) using the QIAsymphony DSP Virus/Pathogen Midi Kit (Qiagen) according to the manufacturer's instructions.

### Library preparation and sequencing

Library preparation was performed using Illumina DNA prep (Illumina, San Diego, California, USA). Sample DNA concentration was measured using a Qubit 2.0 or 3.0 fluorometer (Thermo Fisher, Waltham, MA, USA) before library preparation according to the manufacturer's instructions. Normalisation and pooling were performed dependent on each fragment length, determined using a TapeStation 4200 system (Agilent, Santa Clara, CA, USA), and DNA concentrations were measured with a Qubit 2.0 or 3.0 fluorometer. Whole genome sequencing of the pooled samples was performed on a MiSeq instrument (Illumina) using v2 500 or v3 600 kit (Illumina) according to the manufacturer's instructions.

### Bioinformatic analysis

**Figure 1:**
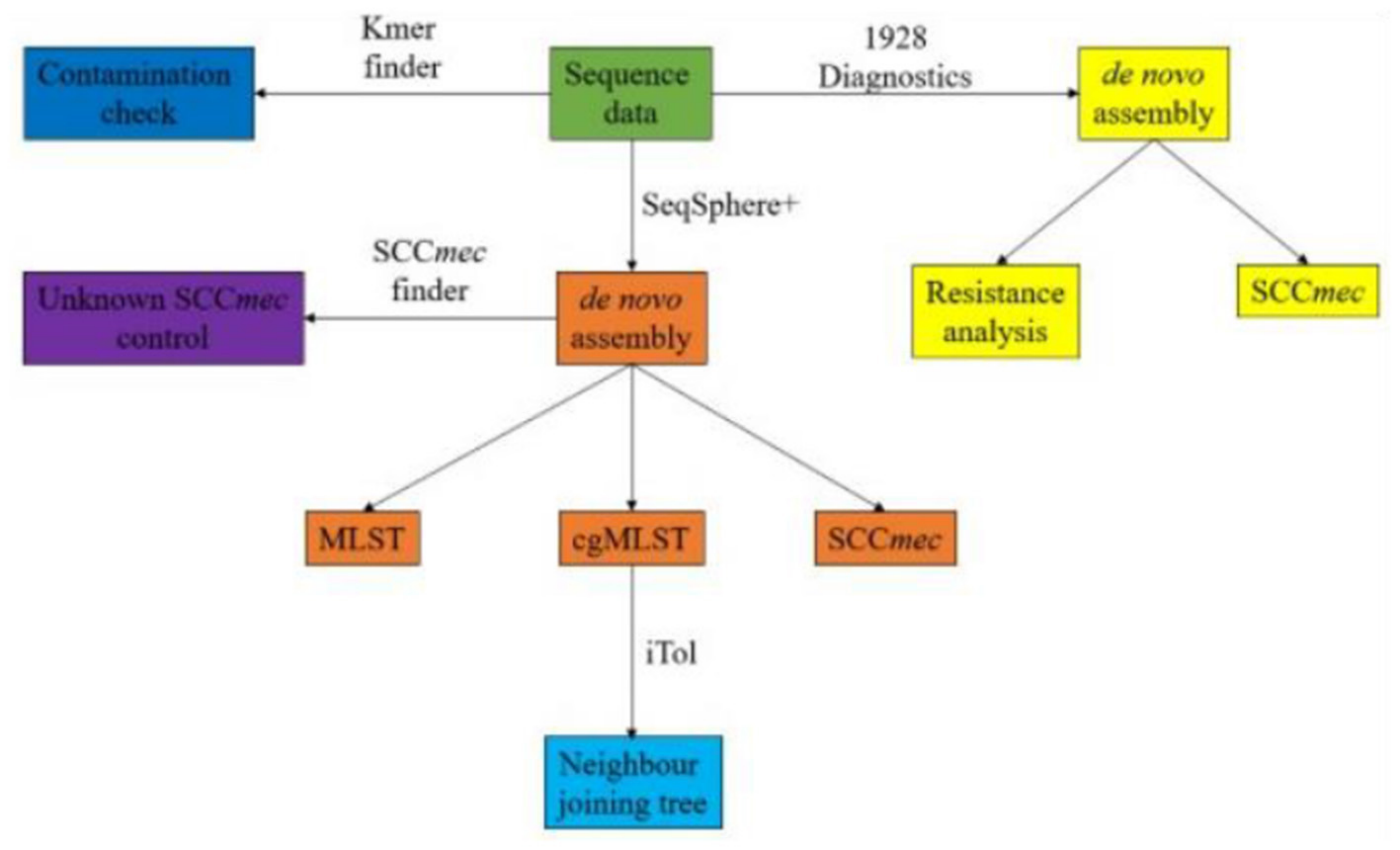
Workflow of bioinformatic analysis from raw sequence data.

An overview of the bioinformatic workflow is shown in Figure 1. Sequence data were processed using the Velvet assembler (version 1.1.04) for *de novo* assembly within the Ridom SeqSphere+ software package (version 8.3.1) (Ridom, Münster, Germany). Multilocus sequence typing (MLST) and core genome MLST (cgMLST) were performed using Ridom SeqSphere+ (Zerbino and Birney, 2008). Each isolate was quality controlled for an average read length of >200 bp, coverage ≥ 50x, and a percentage of good targets for cgMLST of ≥96% using Ridom SeqSphere+. Isolates containing residues of other species were further analysed using KmerFinder to rule out contamination and confirm species identification (Hasman et al., 2014; Larsen et al., 2014; Clausen et al., 2018). For SCC*mec* identification, presence of PVL and the genomic antibiotics resistance analysis, 1928 Diagnostics (version 2022-03.2) (1928 Diagnostics, Gothenburg, Sweden) was used. Genotypic resistance mechanisms were analysed on a gene or point mutation level. Inducible resistance to clindamycin was reported if *ermA* or *ermC* was present (Fiebelkorn et al., 2003; Steward et al., 2005). Isolates were considered positive for PVL if *lukS/F-pv* were found. Isolates were considered USA300 clones on the basis of the following criteria: PVL-positive, arginine catabolic mobile element (ACME)-positive, and ST8/SCC*mec* IV, where PVL and ACME elements were identified using SeqSphere+.

Isolates presenting an unknown SCC*mec* were controlled using SCC*mec* Finder (v.1.2) (Kaya et al., 2018). Neighbour joining trees were constructed in SeqSphere+ and visualised using iTol (version 6.8) (Biobyte Solutions, Heidelberg, Germany) (Letunic and Bork, 2007). Heat maps were constructed using Microsoft Excel (version 2307) (Microsoft, Redmond, Washington, USA). Statistic calculations for comparison between the cohorts were performed with a chi-square test and/or Fisher's exact test using IBM SPSS (version 29.0.0.0) (IBM, Armonk, New York, USA) and R (R Core Team, 2024).

### Ethics

The study was approved by the Ethical Review Authority, Sweden (ref: 2019/06433).

## Results

**Figure 2:**
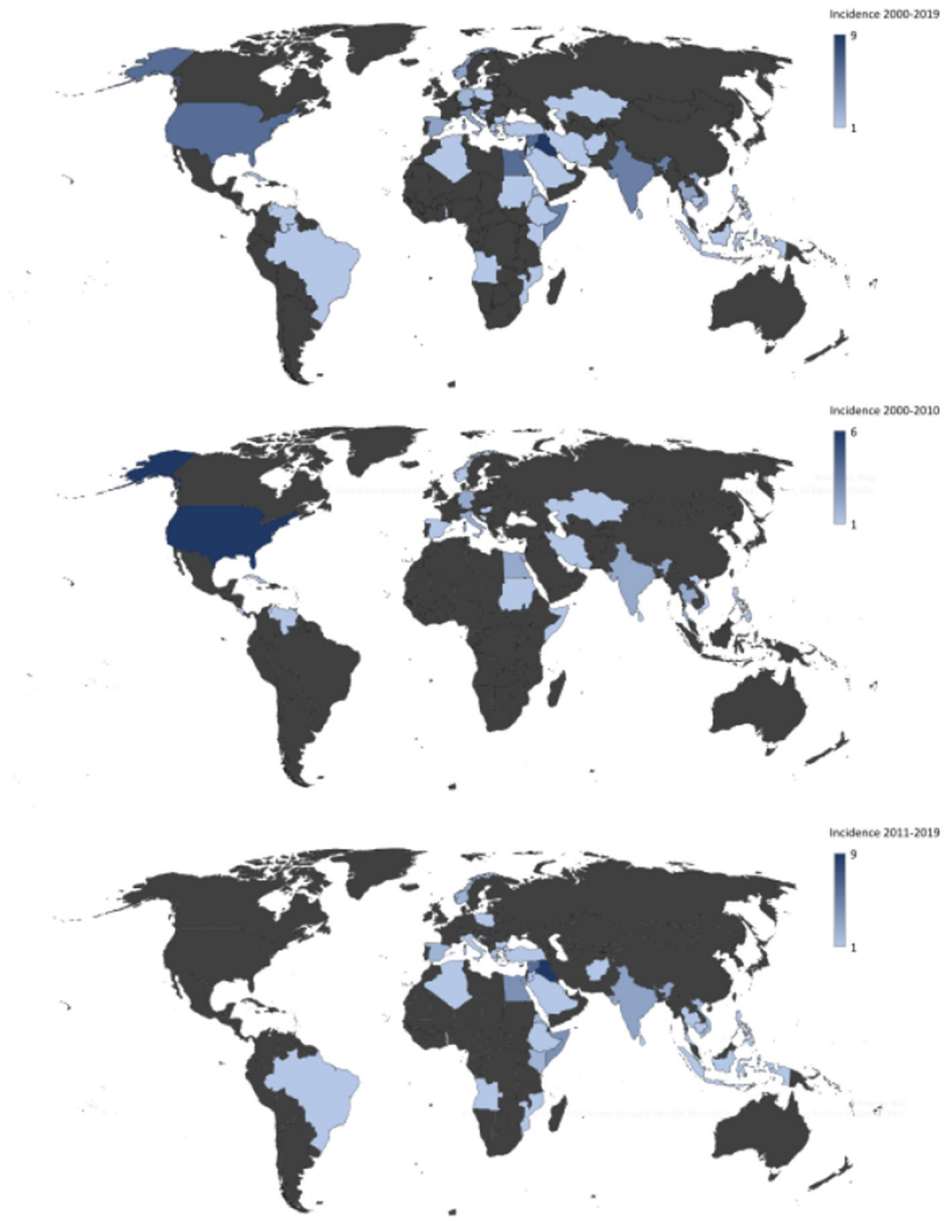
Heat map showing country of origin for cases of community-acquired methicillin-resistant *Staphylococcus aureus* in Region Örebro County during 2000–2019 with presumed origin of infection outside of Sweden.

In total, 330 CA-MRSA isolates were recovered in Region Örebro County during 2000–2019. Four isolates presented possible contamination and/or shared genomic elements, but all were confirmed as *S. aureus* using KmerFinder. The isolates were divided into two cohorts: one with isolates from the years 2000–2010 (*n* = 107) and one with isolates from 2011 to 2019 (*n* = 223). Thus, an increase in reported primary cases of CA-MRSA between the two periods was noted. In 67 cases, the country of acquisition could not be verified: 30 cases (28%) during 2000–2010 and 37 cases (17%) during 2011–2019. There was an increase in the number of isolated CA-MRSA with an origin in the Middle East, with only two cases (2%) during 2000–2010 compared to 20 cases (9%) during 2011–2019 (*p* = 0.017), and a decrease in the number of cases originating in the United States, with six isolates from 2000–2010 and none from 2011 to 2019 (*p* ≤ 0.005) (Figure 2).

*mecA* was present in 328/330 (99.3%) isolates and *mecC* in 2/330 (0.6%) with ST130 and SCC*mec* type XI. All *mecA-*positive isolates displayed resistance to isoxazolyl-penicillin by routine antibiotic susceptibility testing (screening with a cefoxitin disc), while 308/330 (93%) isolates were found to be positive for *blaZ* and thus assessed as resistant to penicillinase-labile penicillin. No genotypic resistance to mupirocin was found. In comparison to the earlier cohort, the later cohort showed increased genotypic resistance to trimethoprim (12.1% vs. 24.7%), (*p* = 0.009), increased induced clindamycin resistance (12.1% vs. 21.1%) (*p* = 0.066), and decreased resistance to fusidic acid (23.4% vs. 16.6%) (*p* = 0.175). Genotypic resistance to ciprofloxacin, erythromycin, gentamicin, rifampicin, and tetracycline remained relatively stable over time (Table 1).

**Table 1:** Predicted antibiotics resistance.

	**2000–2010 (*****n*** = **107)**		**2011–2019 (*****n*** = **223)**		**Difference**	***p*-value**
	***n*=**	**%**	***n*=**	**%**		
Ciprofloxacin	21	19.6%	38	17%	−2.6%	*p* = 0.672
Clindamycin	1	0.9%	10	4.5%	3.5%	*p* = 0.195
Clindamycin (inducible)	13	12.1%	47	21.1%	8.9%	*p* = 0.066
Erythromycin	29	27.1%	60	26.9%	−0.2%	*p* = 1.000
Fucidic acid	25	23.4%	37	16.6%	6.8%	*p* = 0.175
Gentamicin	11	10.3%	29	13.0%	2.7%	*p* = 0.596
Rifampicin	2	1.9%	2	0.9%	−1.0%	*p* = 0.827
Tetracycline	37	34.6%	70	31.4%	−3.2%	*p* = 0.013
Trimethoprim	13	12.1%	55	24.7%	12.5%	*p* < 0.005
Mupirocin	0	0%	0	0%	0%	-

Genotypic predicted antibiotic resistance of community acquired methicillin-resistant *Staphylococcus* aureus in Region Örebro County during 2000–2019 divided into two cohorts, based on whole genome sequencing on 330 isolates on an Illumina platform. 228 isolates carried *mecA* and two carried *mecC*.

**Figure 3:**
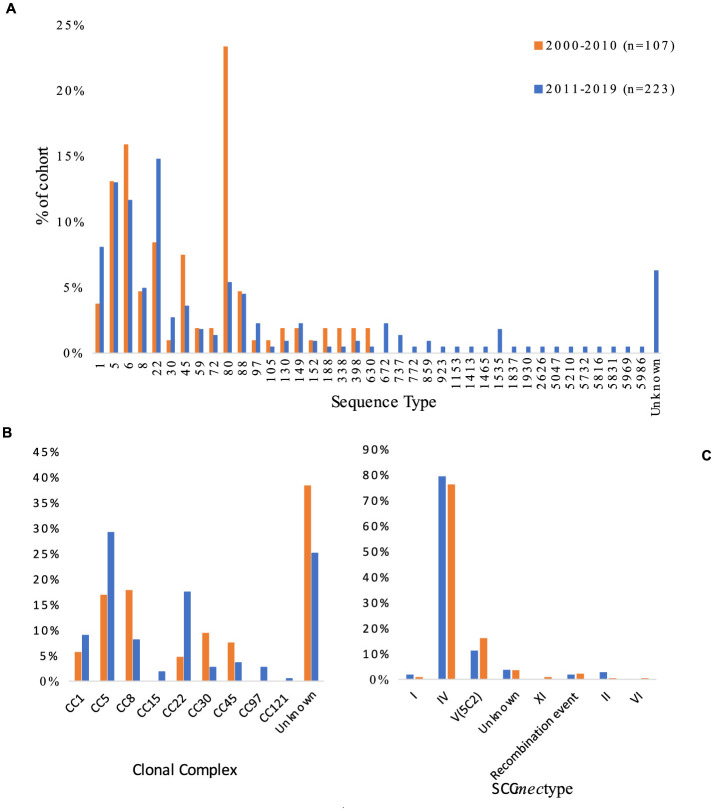
Variation in **(A)** sequence type, **(B)** clonal complex, and **(C)** SCC*mec* among 330 isolates of primary cases of community-acquired methicillin-resistant *Staphylococcus aureus* collected in Region Örebro County during 2000–2019. Recombination events are defined as isolates that showed presence of some SCC*mec* genes and other recombinase complexes, indicating a recombination event.

**Figure 4:**
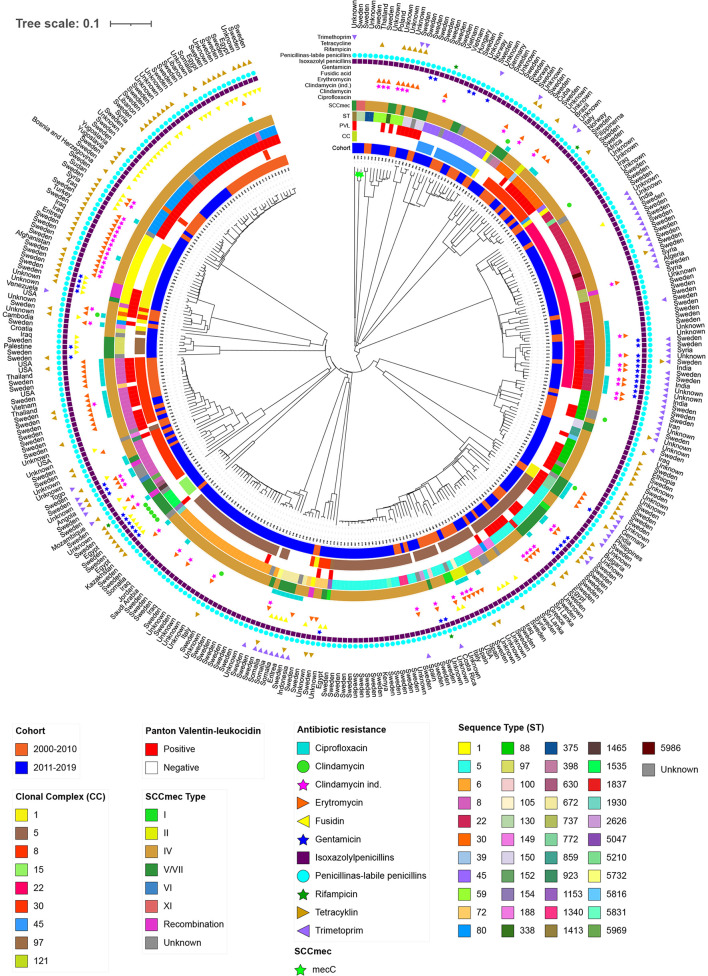
Phylogenetic neighbour joining tree using 1,861 core genome multilocus sequence typing (cgMLST) genes of isolates from primary cases of community-acquired methicillin-resistant *Staphylococcus aureus* in Region Örebro County during 2000–2019, divided into two cohorts: 2000–2010 (*n* = 107) and 2011–2019 (*n* = 223). Each leaf represents an isolate. Scale presents the percentage difference between the 1,861 cgMLST genes analysed.

Analysis of SCC*mec* showed type IV to be the most common in both cohorts, followed by type V (5C2) (Figure 3). However, the prevalence of type IV showed a slight decrease over time, with a difference of 3.2% between the cohorts (*p* = 0.62). SCC*mec* types I and II also showed slight decreases in prevalence, with between-cohort differences of 1% (type I) and 2.4% (type II). Moreover recombination events remained relatively stable between the cohorts, but the 2011–2019 cohort saw the introduction of SCC*mec* types VI and XI, both acquired in Sweden. The only SCC*mec* type to increase in prevalence during the study period was V/VII, with an increase of 4.9% between the cohorts. The 2000–2010 cohort had 4/107 (3.7%) isolates with unknown SCC*mec* type (*SCCmec* genes were present but identification was not possible) and 2/107 (1.8%) isolates with indications of recombination events (presence of some SCC*mec* genes and other recombinase complexes). The 2011–2019 cohort had 8/223 (4%) isolates with unknown SCC*mec* type and 5/223 (2%) with indications of possible recombination events (Figure 4).

MLST analysis showed that ST80 (23%) was the dominant ST during 2000–2010 and ST22 (15%) was dominant during 2011–2019, but both cohorts presented heterogenic diversity. Analysis of CCs was inconclusive due to the high number of unknown CCs found, but the largest increase in prevalence was seen for CC22 (13%) (*p* < 0.005) followed by CC5 (12%), and the largest decrease in prevalence was seen for CC8 (−10%) (*p* = 0.015). Combined data of origin and CC showed that 11/223 (5%) of the isolates from 2011 to 2019 belonging to CC5 or CC22 had an origin in the Middle East, compared to 1/107 (1%) for the CC5/CC22 isolates from 2000 to 2010.

ST and SCC*mec* showed 24 different combinations; ST 80/SCC*mec* IV (23%) was the most common during 2000–2010, and ST 22/SCC*mec* IV (14%) was the most common during 2011–2019. Between 2000–2010 and 2011–2019, the incidence of ST80/SCC*mec* IV decreased by 18% (p ≤ 0.005) and the incidence of ST8/SCC*mec* IV decreased by 12% (p ≤ 0.005), while the incidence of ST22/SCC*mec* IV increased by almost 10%, yet the increase was not significant (*p* = 0.16). Of the 16 isolates with ST8/SCC*mec* IV in the 2000–2010 cohort, 12 were identified as USA300 clones; only one such clone was seen in the 2011–2019 cohort. ST6 did not appear in the 2000–2010 cohort at all, but the 2011–2019 cohort included 26 isolates (12%), of which six were acquired in the Middle East or Africa and one in Italy. A 30% decrease in the prevalence of PVL among CA-MRSA was observed between the two periods (*p* ≤ 0.005), though the number of reported cases remained constant (2000–2010: *n* = 62, 2010–2019: *n* = 63).

Phylogenetic analysis based on 1,861 cgMLST genes showed major clusters with a combination of isolates from both cohorts. However, one major (CC5) and three minor clusters (CC15, CC97, and unclassified) contained isolates exclusively from 2011 to 2019. No clusters presented homogeneity regarding country of origin, except minor clusters of 2–3 isolates (Figure 4).

## Discussion

The county is considered to be of low endemicity, and on an international level Sweden is similar to the other Nordic countries (ECDC, 2024). The trends in Norway and Denmark during this period is an increase in prevalence of CA-MRSA as can be seen in Region Örebro County as well (Petersen et al., 2021). Region Örebro County consists of a mix of urban, suburban, and rural areas covering 8,500 km^2^. It has approximately 300,000 inhabitants, and an increase of 12% in its population between 1998 and 2023 was noted. Berglund et al. (2005a) identified 29 clinically relevant cases of CA-MRSA during 1987–2004 from a selection of 57 cases of MRSA, with ST80 and ST45 being the dominant STs; ST80 was exclusively found in CA-MRSA while ST45 was found among both HA-MRSA and CA-MRSA. This is consistent with a more recent finding of ST80 as being the dominant clone of CA-MRSA in Europe (Tristan et al., 2007; Brauner et al., 2013; Drougka et al., 2014). The present study shows an alteration in the genomic characteristics of CA-MRSA in Region Örebro County during 2000–2019; ST80 dominated during 2000–2010, as could be seen in northern Norway in the early 2000s, but in 2011–2019 the distribution shifted towards ST22, closely followed by ST5 and ST6. The combination of STs and SCC*mec* also showed a shift in dominance, from ST80/SCC*mec* IV in 2000–2010 to ST22/SCC*mec* IV in 2011–2019 (Hanssen et al., 2005). We found 12 SCC*mec* elements that could not be classified, three of which clustered together. These three elements all showed a complex resistance pattern which had more in common with the phylogenetic tree neighbouring SCC*mec* type V(5C2) isolates. Also, in the phylogenetic tree, neighboured by isolates carrying SCC*mec* V/VII and VI, indicating that this cluster might be a novel subtype or recombination.

The present results indicate that the SCC*mec* characteristics remains quite stable over longer periods of time and might not be affected by demographic shifts as much as the ST appears to be, however, if the appearance of stability is due to one or more local clones and/or an influx of one or multiple clones with other SCC*mec* type IV elements is unknown. As phage transduction has been considered a candidate for horizontal gene transfer of short SCC*mec* elements, and the SCC*mec* type IV element appears to have a lower fitness cost, it seems possible that the prevalence of the SCC*mec* type IV element, strains carrying this element might increase and continue to invade hospital and healthcare settings, along with a continued increased transductal circulation in the community (Lee et al., 2007; Scharn et al., 2013). Other studies in Malaysia, Ireland, Australia, and Germany have also found SCC*mec* type IV to be the dominant SCC*mec* type, indicating that this SCC*mec* type has found a favourable balance in its survivability (resistance mechanism and fitness cost) and ease of transmission (element size and virulence factors). This warrants further research into what mechanisms contribute to its success, in order to prepare to contain future possible outbreaks (Coombs et al., 2014; Shore et al., 2014; Monecke et al., 2016; Nor Amdan et al., 2024).

The decrease in prevalence of PVL-positive isolates from the first cohort to the second cohort follows the general trend in both Norway and Denmark of a decreasing incidence of PVL positive isolates, ST8 and ST80, containing SCC*mec* type IV, isolates normally associated with PVL seems to follow the PVL trend (Petersen et al., 2021; Rønning et al., 2024). In addition, three isolates that were reported to have been contracted in the United States during 2000–2010 were identified as USA300 clones (ST8/SCC*mec* IV, PVL and ACME positive) (Diep et al., 2006; Otter and French, 2010). These findings follow the combined decrease of ST8/SCC*mec* IV and ST80/SCC*mec* IV, indicating that the high PVL incidence (58%) during 2000–2010 in Region Örebro County might be partly linked to these ST/SCC*mec* combinations, as several of the emerging ST22 isolates during 2011–2019 were PVL-negative. An Italian study covering all MRSA cases in a paediatric hospital during 2013–2015 found a 27.8% prevalence of PVL-positive isolates; however, other studies report significant variations in PVL prevalence, hence no conclusion of the present study's results regarding PVL prevalence could be made (Berktold et al., 2012; Shore et al., 2014; Manara et al., 2018).

ST22/SCC*mec* IV was assumed to be one of the major CA-MRSA types in Europe and in the Middle East in 2018. In this study we found ST22 isolates originating from India which might be the same clone presented by Abrudan et.al in a recent study strengthening the assumed migration via travel (Abrudan et al., 2023). ST6/SCC*mec* IV has also been described as an emerging clone in the Middle East and was one of the most common combinations in a hospital in Oman during 2011 (Udo et al., 2014). In this case, it is possible that ST6 has been introduced to Region Örebro County via migration or other travel, as at least 8 (31%) and perhaps as many as 14 of the 26 ST6 isolates were acquired abroad. Sweden's population was approximately 9.5 million in 2010, and 10.4 million in 2020. Migration to Sweden during 2010–2019 included ~1.2 million people, 250,000 of whom immigrated from the Middle East (Statistics Sweden, n.d.). Almost 450,000 people immigrated from other parts of Europe and another 520,000 from Asia, which are other areas where ST22/SCC*mec* IV is commonly found. However, none of the foreign-acquired ST22 isolates found in this study originated in Europe; instead, the ST22 isolates from 2010–2019 originated in India and in the Middle East. Additionally, four ST6 isolates originated in the Middle East, also from the 2010–2019 period, concurring with an earlier study confirming the presence of ST6 in that region (Udo et al., 2014). In addition, other isolates with an origin outside of Sweden originated in countries reported as being routes for refugees, for example Egypt and Italy. Bartels et al. have also confirmed the introduction of ST6 in Denmark which could be associated to the clone found by Udo et.al, raising the possibility that the ST6 isolates found in Region Örebro County might have the same origin, however this needs further research to be confirmed (Bartels et al., 2015).

These findings might indicate a connexion between migration to Sweden from the Middle East and Africa and the change in ST22/SCC*mec* IV becoming the dominant type, connected to the increased prevalence of STs belonging to CC5 and CC22. This is in accordance with the findings of Rønning et.al in Denmark (Rønning et al., 2024). The USA300 clone seems to have failed in establishing a major presence in Region Örebro County; it perhaps has been outcompeted by STs assigned to CC5 and CC22, as only one isolate in the late cohort could be identified as USA300 compared to 12 in the early smaller cohort (Bouchiat et al., 2017). Although the migratory flow from the Middle East has probably affected the *S. aureus* epidemiology in Europe, the full aspect of the CA-MRSA epidemiology in general is still an unclear subject due to asymptomatic carriers, and individuals with uncomplicated skin and soft tissue infections reluctant to seek medical care, or presence of possible non-human reservoirs, as can also be suspected in this study with the findings of isolates of ST398, otherwise associated with livestock associated MRSA (Figure 4) (Lee et al., 2007; Normanno et al., 2020).

The absence of resistance towards mupirocin indicates that effective eradication of nasal carriage among healthcare workers and patients is still possible in Region Örebro county. Several of the individuals found carrying CA-MRSA are asymptomatic as the Communicable Diseases Act governs mandatory contact tracing and a “Search and Destroy” policy in case of newly discovered cases with confirmed MRSA. Most isolates presenting a resistance towards clindamycin was regarded as inducible clindamycin resistance. However, almost 5% of the isolates from 2011 to 2019 showed a constitutive resistance towards clindamycin, compared to 1% during 2000–2010, presenting a possible trend that should be supervised.

Limitations of this study include the geographic and demographic constraints. The study focuses exclusively on Region Örebro County, which may limit the generalizability of the findings to other regions or countries with different demographic compositions and healthcare infrastructures. Comparison with other Nordic countries (e.g., Norway and Denmark) give some strength to the findings, however, some of these studies, as well as reports from Statens Serum Institut, Copenhagen, Denmark, are mainly based on *spa-*typing making direct comparison difficult as this study is based on whole genome sequencing MLST, but general trends might still be possible to follow. In addition, the division of isolates into two distinct cohorts (2000–2010 and 2011–2019) might have obscured subtle changes and trends occurring within shorter timeframes, and the lack of data on country of origin for 20% of the cases limits the ability to draw comprehensive conclusions about the impact of immigration and international travel on the epidemiology of CA-MRSA.

In conclusion, this study provides insights into the epidemiology and genomics of CA-MRSA and the possible impact of migration in Region Örebro County, Sweden. By analysing 330 isolates over two decades, we identified shifts in SCC*mec* types and resistance profiles. The surveillance of resistance genes, identification of unknown SCC*mec* elements, and the shift of prevailing ST types, some likely caused by migration or travel, highlights the evolving nature of MRSA and underscores the need for ongoing surveillance and genomic monitoring. Despite limitations, our findings emphasise the importance of adapting public health strategies to effectively address the dynamic landscape of MRSA infections.

## Data Availability

The datasets presented in this study can be found in online repositories. The names of the repository/repositories and accession number(s) can be found below: https://www.ebi.ac.uk/ena, PRJEB77022.
